# ING Tumour Suppressors and ING Splice Variants as Coregulators of the Androgen Receptor Signalling in Prostate Cancer

**DOI:** 10.3390/cells10102599

**Published:** 2021-09-29

**Authors:** Anna Melekhova, Aria Baniahmad

**Affiliations:** Institute of Human Genetics, Jena University Hospital, 07740 Jena, Germany; melekhova.anna@gmail.com

**Keywords:** inhibitor of growth family, tumour suppressor type 2, androgen receptor, prostate cancer, corepressor, coactivator, epigenetic reader, splice variants, isoforms, cellular senescence, epithelial-mesenchymal transition

## Abstract

Prevention and overcoming castration resistance of prostate cancer (PC) remains one of the main unsolved problems in modern oncology. Hence, many studies are focused on the investigation of novel androgen receptor (AR) regulators that could serve as potential drug targets in disease therapy. Among such factors, inhibitor of growth (ING) proteins were identified. Some ING proteins act as AR transcriptional coregulators, indicating their relevance for PC research. The ING family consists of five protein-coding genes from *ING1* to *ING5* and pseudogene *INGX*. The *ING* genes were revealed through their sequence homology to the first identified *ING*1 from an in vivo screen. ING factors are a part of histone modification complexes. With the help of the conserved plant homeodomain (PHD) motif, ING factors bind to Histone 3 Lysine 4 (H3K4) methylation mark with a stronger affinity to the highest methylation grade H3K4me3 and recruit histone acetyltransferases (HAT) and histone deacetylases (HDAC) to chromatin. ING1 and ING2 are core subunits of mSIN3a-HDAC corepressor complexes, whereas ING3–5 interact with different HAT complexes that serve as coactivators. ING members belong to type II tumour suppressors and are frequently downregulated in many types of malignancies, including PC. As the family name indicates, ING proteins are able to inhibit cell growth and tumour development via regulation of cell cycle and cancer-relevant pathways such as apoptosis, cellular senescence, DNA repair, cell migration, invasion, and angiogenesis. Many ING splice variants that enhance the diversity of ING activity were discovered. However, it seems that the existence of multiple ING splice variants is underestimated, since alternative splice variants, such as the AR coregulators ING1 and ING3, counteract full-length ING and thus play an opposite functional role. These results open a novel prospective investigation direction in understanding ING factors biology in PC and other malignancies.

## 1. Introduction

### 1.1. ING Expression Levels in PC

ING proteins have the property of type II tumour suppressors in diverse cancer types. They were widely investigated in recent years for their implication as possible PC biomarkers serving for disease prognosis prediction. For instance, ING1 expression levels are downregulated in castration-resistant PC cells in comparison to an androgen-sensitive cell line [1]. ING4 expression is lost in more than a half of human primary prostate malignancies [2]. Moreover, ING4 loss is tightly correlated with loss of another tumour suppressor, PTEN, and associated with a dysfunctional differentiation program of basal epithelial cells resulting in prostate tumorigenesis [2,3]. Both malignant tissues isolated from PC patients and PC cell lines demonstrate decreased ING5 expression when compared to the non-malignant control [4]. In contrast, higher ING3 levels were associated with poor disease prognosis in a subgroup with low AR and higher metastatic potential, specifically for PC patients [5,6,7]. 

Similar to PC, ING proteins show reduced expression in many other malignancies. Decreased levels of ING1, loss of heterozygosity, or mutations in *ING1* were detected in breast cancer, melanoma, head carcinoma, and astrocytoma [8,9,10,11]. *ING2* expression is reduced in hepatocellular carcinoma and lost in lung carcinoma [12,13]. ING3 is downregulated in head and neck carcinoma, melanoma, and hepatocellular carcinoma [14,15,16], while ING4 is reduced in bladder and colorectal cancer [17,18]. ING5 downregulation was observed in oral squamous cell carcinoma and lung cancer [19,20]. 

Interestingly, not only the expression level, but also cellular localisation of ING proteins is correlated with poor patients’ survival [21,22,23]. For example, when ING3 protein accumulates in the nucleus, it suppresses melanoma cell migration, invasion, and angiogenesis [24]. In contrast, ING3 translocation into cytoplasm induces tumorigenesis and is associated with tumour progression in head and neck squamous cell carcinoma [14]. This suggests that not only expression levels, but also subcellular localization is an important factor for ING activity.

Taken together, ING factors are often downregulated in various cancer types associated with poor patients’ survival. Mouse knockout models supported tumour suppressor role of ING proteins. *Ing1*-deficient mice frequently developed B-cell lymphomas and *Ing2*-deficient mice showed higher numbers of soft tissue sarcomas in males [25,26]. *Ing3* deficiency in mice is early embryonal lethal, and thus, the role in cancer remains unclear [27]. In contrast to *Ing1* and *Ing2*, *Ing4* knockout in mice did not result in tumorigenesis [28].

### 1.2. Tumour Suppressive Pathways of ING Factors

On a molecular level, one of the tumour suppressive functions of ING proteins is their ability to induce cell death. *ING4* delivered by adenovirus into PC cell line PC3 was reported to significantly inhibit cell proliferation and promote apoptosis by inducing *TP*53, *BAX*, *Caspase-3* expression and reducing *BCL2* expression [29]. ING5 reduced PC cell growth through apoptotic cell death induction in both androgen-dependent (LNCaP) and androgen-independent (PC3) human cell lines [4]; however, the exact mechanisms of ING member-induced apoptosis were explored in more detail in other cancer types. ING1 was reported to enhance p53-dependent apoptosis rate in melanoma cells after UVB irradiation by upregulation of pro-apoptotic Bax expression and changed mitochondrial membrane potential [30]. ING3 promoted apoptosis upon UV irradiation independent from p53 through Fas/caspase-8 pathway along with an increased cleavage of Bid and caspases-8, -9, and -3 [31]. ING4 overexpression in A375 cells led to similar effects as ING3, resulting in induced levels of Fas, cleaved caspase-8 and caspase-3, and decreased levels of FasL followed by apoptosis induction [32].

On the other hand, ING proteins can restrict malignant cells growth by inducing cellular senescence, an irreversible cell cycle arrest in G0/G1 cell cycle phase [1,33,34,35,36,37].

In general, ING factors are required for many cell types to activate cellular senescence and their absence abolishes this process. For example, RNA-interference-mediated ING1 suppression in human fibroblasts resulted in bypassing oncogene-induced senescence [34]. This is consistent with the data concerning the role of ING proteins in cell cycle arrest induction in PC. The ectopic expression of ING1 in PC cell line led to upregulated expression of the cell cycle inhibitors p16, p27, induction of cellular senescence and consequently to inhibition of proliferation [1]. Interestingly, in PC cells, cellular senescence is induced by low levels of ING1 through a compensatory feedback loop mechanism, since ING1 knockdown induced ING2 expression [38]. ING2, in turn, increased the levels of cell cycle inhibitor p16 and senescent PC cells [38]. Both ING3 up- and downregulation in PC cells resulted in cellular senescence induction through the upregulation of p21 expression [39]. This suggests that the ING3 protein levels in PC must be balanced in order to allow cancer cells to proliferate. Notably, the expression of ING3 splice variants must also be taken into consideration since they may affect intracellular pathways in a distinct manner [39].

### 1.3. ING Factors Regulate EMT

Some ING proteins were reported to hamper angiogenesis, migration, and invasion capacity of malignant cells [17,24,40,41,42,43]. ING3 protects PC cells from metastasis-associated epithelial–mesenchymal transition (EMT) characterised by decreased protein expression of epithelial marker E-Cadherin and upregulated expression of mesenchymal markers N-Cadherin and Vimentin [39,44,45]. ING4 and ING5 possess high amino acid sequence homology and share inhibitory function on EMT that subsequently reduce migration and invasion capacity of malignant cells [18,46,47,48,49,50]. ING4 was reported to negatively regulate cell proliferation (hepatocellular carcinoma) through the NF-κB/miR-155 pathway, upregulation of nuclear levels of FOXO3a and its enhanced transcriptional activity [51]. ING4 also regulates EMT. ING4 overexpression in both in vivo and in vitro experiments significantly reduced hepatocellular carcinoma cell growth through cell cycle G1 phase arrest and apoptosis, inhibition of migration, and invasion [51]. Furthermore, overexpression of ING4 in HK2 cells repressed EMT induction upon hypoxia [52]. In addition, studies concerning lung cancer showed that ING5 knockdown-induced EMT was abolished upon treatment of cells with WNT/β-catenin inhibitor, suggesting that ING5 inhibits EMT via WNT/β-catenin signalling [49]. ING5 hampered growth and invasion of oesophageal squamous cell carcinoma in vitro and suppressed metastasis in vivo by the AKT pathway [42]. Accordingly, ING5 also inhibits EMT since the loss of ING5 expression in lung cancer enhanced migration and invasion through activation of EGFR/PI3K/AKT and IL-6/STAT3 signalling pathways, which led to the induction of EMT [50].

Altogether, ING family members act as tumour suppressor type II in various cancer types, including PC, repress EMT, inhibit tumour cell proliferation and metastatic potential via various cellular mechanisms.

## 2. ING Proteins as Coregulators of AR

Recent studies identified that some ING proteins serve as coregulators of the AR with additional relevance for PC research [1,6,38]. The growth and development of both normal and malignant prostate rely on AR signalling [53]. Modern therapies against PC include androgen-deprivation therapy (ADT) and the treatment with antiandrogens as blockers of the AR pathway [54,55]; however, after a few months to up to few years of treatment, tumours become castration-resistant and do not respond to antiandrogens therapy. Nevertheless, AR signalling remains active in castration-resistant PC [56,57]; therefore, the investigation of AR coregulators is crucial to elucidate the regulation of AR signalling.

ING1 and ING2 were both identified as AR-corepressors [1,38]. Based on the results from co-immunoprecipitation, GST-pull-down, and quantitative intracellular colocalization in vitro and surface-enhanced laser desorption ionization-mass spectrometry (SELDI-MS) in combination with immunological techniques in vivo, ING1 was shown to physically interact with AR in the nucleus upon treatment of cells with androgens [1]. Moreover, ING1 inhibited the expression of AR-responsive genes in PC cells, while the *Ing1*-knockout mouse exhibited enhanced AR transcriptional activity, supporting the hypothesis of an AR-corepressor role of ING1.

**Figure 1:**
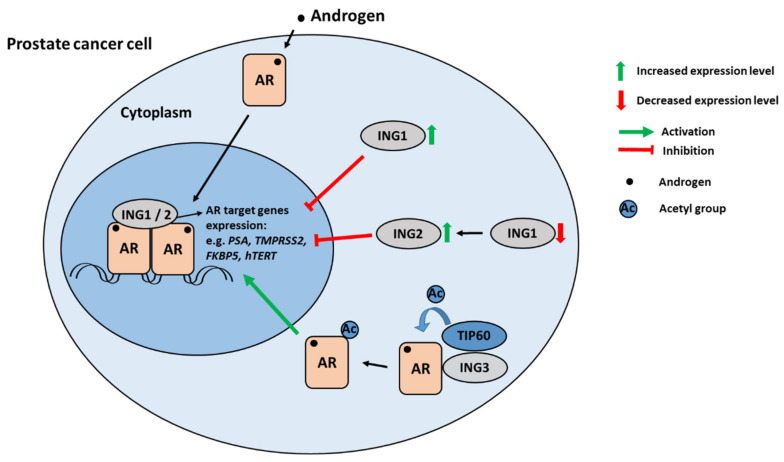
Schematic view of ING factors as coregulators of AR in PC. Androgens (black circle) bind to AR that translocates into the nucleus. ING1 and ING2 proteins interact with AR and serve as AR corepressors leading to reduced AR target gene expression of positively regulated AR target genes. Furthermore, ING1 and ING2 mediate the transrepression of the AR silenced target gene, *hTERT* [58]. On the other hand, ING3 acts as AR-coactivator, as it recruits Tip60 to AR that becomes acetylated (Ac), leading to enhanced transactivation.

Interestingly, since the related pair ING1 and ING2 is highly homologous, in ING1-deficient condition, ING2 displayed compensatory function by hampering AR [38]. In detail, the knockdown of ING1 resulted in increased ING2 protein levels that led to PC cells’ growth retardation accompanied by repressed AR-mediated gene expression (Figure 1). This indicates a feedback mechanism to compensate for the loss of ING1 activity by ING2 and suggests to co-analyse both ING1 and ING2 levels in the same PC tissues. Co-immunoprecipitation experiments showed that ING2 also physically binds to AR and androgens promoted this interaction [38]. This finding was further supported by in vivo experiment, where prostate-specific target genes of AR were reduced in *Ing1* knockout mice. This indicates that both ING1 and ING2 are ligand-dependent corepressors of AR (Figure 1).

Each ING1 and ING2 can repress androgen-induced expression of another positively regulated AR target gene *KLK3*. *KLK3* encodes the prostate-specific antigen (PSA) and serves as an important diagnostic marker for PC. Additionally, the direct AR target genes induced by androgens, *TMPRSS2* and *FKBP5* are repressed by ING1 and ING2 overexpression [1,38]. Recently, the negatively regulated AR target gene *hTERT* was shown to be mediated by ING1 and ING2 [58]. *hTERT* encodes the catalytic subunit of the telomerase and is a tumour promoting factor to avoid telomere shorting. Interestingly, at supraphysiological androgen levels, used in bipolar androgen therapy to treat PC patients [59], *hTERT* expression is repressed by AR. This indicates that AR also has tumour suppressive activity. In line with this observation, the AR, ING1, and ING2 are recruited to the *hTERT* promoter in an androgen-induced manner. Knockdown experiments indicate that ING1 and ING2 mediate the transrepression by AR in both androgen-sensitive and castration-resistant PC cells [58]. These findings suggest that AR acts in concert with ING1 and ING2 to regulate the expression of genes in a tumour suppressive manner.

In contrast to ING1 and ING2, several publications demonstrated that ING3 acts as an oncoprotein in PC and as a coactivator of AR [5,6,7]. ING3 physically binds to AR with the help of its DNA-binding domain (DBD) in a DNA-independent manner. Despite much higher ING3 levels in the nucleus, according to the co-immunoprecipitation results, it suggests that the ING3-AR interaction takes place in the cytoplasm [6]. The interaction promotes the HAT-Tip60-mediated acetylation of AR and coactivator function. The ING3-AR interaction led to AR stabilization, enhanced nuclear translocation, and expression of AR-regulated genes such as *PSA*, *TMPRSS2*, and *FKBP5* [6]. In summary, some ING proteins act as AR coregulators by directly binding to AR and modulating its transcriptional activity.

## 3. INGs Expression Profile in Benign Prostate Tissue and Malignant Prostate Tissue

Surprisingly, according to the dataset from 152 normal prostate tissue samples and 492 PC samples from the GEPIA database and dataset from 52 normal prostate tissue samples and 497 PC samples from UALCAN, ING1-ING5 expression showed no significant difference in the PC group in comparison to non-tumour prostate tissue (Supplemental Figure S1). Additionally, GEPIA and UALCAN survival plots showed no significant correlation between the high expression level of ING factors and poorer patients’ survival (Supplemental Figure S2). These data are not consistent with previous results demonstrating an increased ING3 expression level in aggressive PC and decreased ING4 and ING5 expression in PC [2,4,5].

**Figure 2:**
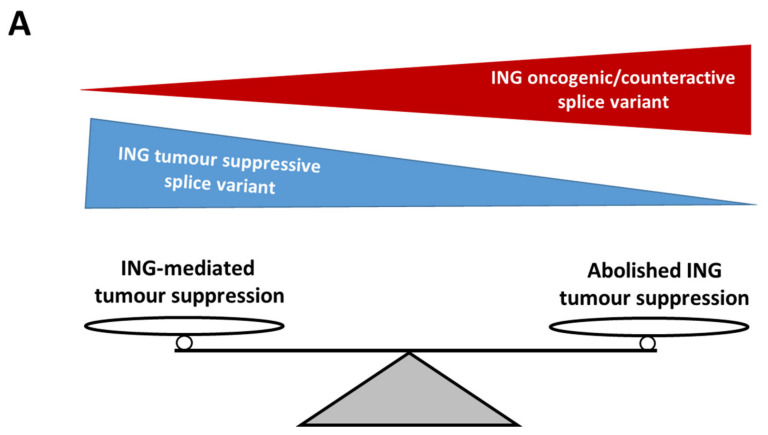

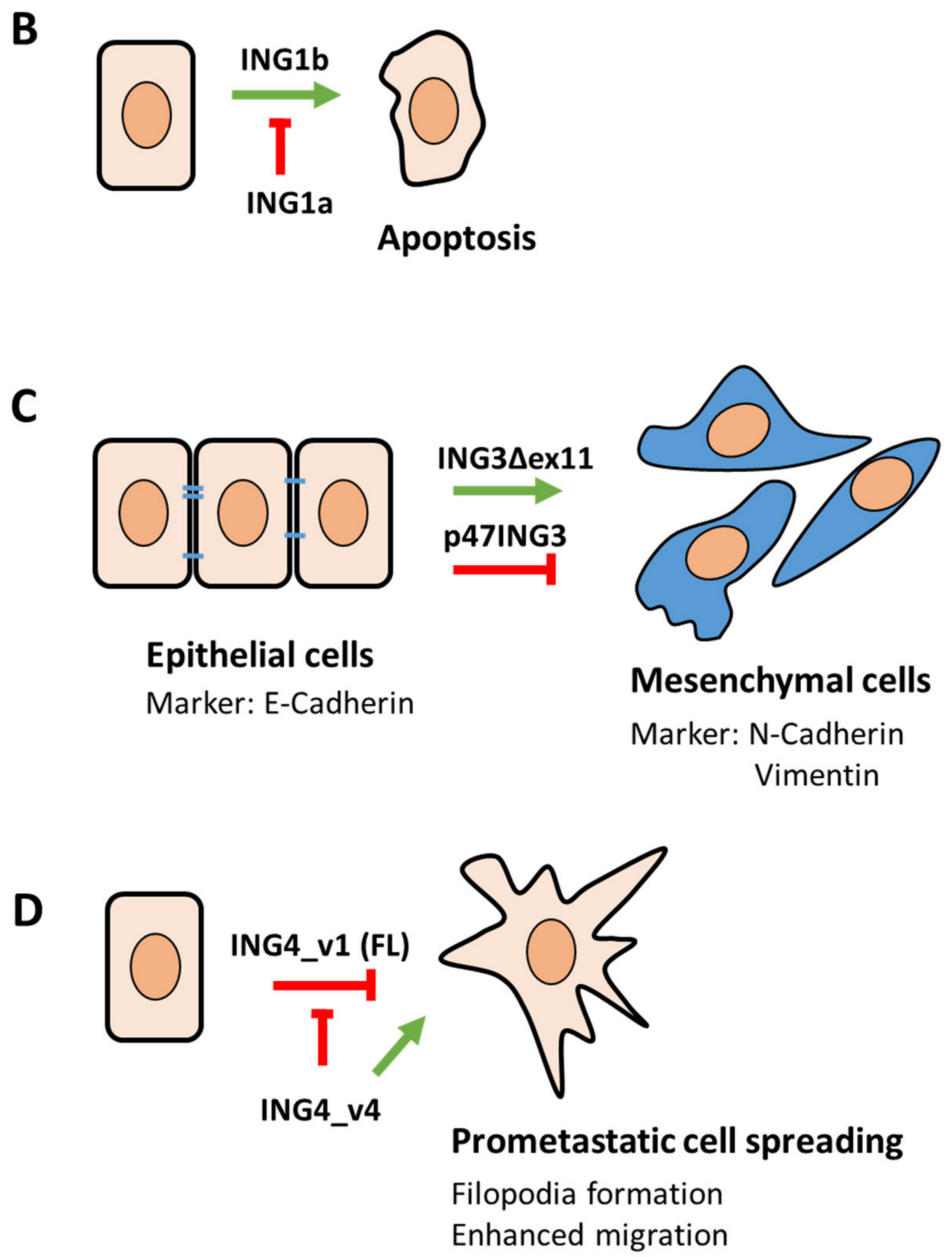
The balance of different ING splice variants expression determines the effect on tumour cells. (**A**) When the expression proportion of ING tumour suppressor isoform increases, ING-mediated tumour suppression is activated in tumour cells. It negatively regulates cell proliferation, migration, and invasion. If ING splice variants equilibrium is shifted to the side of oncogenic/counteractive ING isoforms, tumour suppressive effect is abolished. ING splice variants can functionally counteract in tumour cells. (**B**) ING1a inhibits ING1b-induced apoptosis. (**C**) p47ING3 (full-length) acts as a tumour suppressor and inhibits EMT keeping PC cells in an epithelial state. ING3Δex11 that lacks PHD motif induces EMT in PC cells. (**D**) ING4_v1 (full-length) inhibits premetastatic cell spreading, filopodia formation in malignant cells. ING4_v4 lacking NLS totally abolishes ING4_v1 (full-length)-mediated inhibition of filopodia formation.

One of the probable reasons correlating ING expression with PC could be that expression analyses would benefit if data were derived from stratified patient samples analysing a subgroup with low AR and/or more aggressive cancer stage. Additionally, such studies do not reflect ING protein posttranslational modifications, cellular localization, and ING protein expression levels in cells that may affect ING function in tumour cells. Another possible reason is the existence of ING splice variants and isoforms that usually are not considered separately in analysing expression changes in many studies. Frequently, some splice isoforms cannot be technically detected or distinguished from one another due to experimental limitations, e.g., antibody/primer specificity in various analysis methods such as Western blotting, qRT-PCR, immunohistochemistry, and microarrays. This may lead to contradictory results as different expression studies analyse different sets of ING splice variants as a sum and do not measure the splice variants and isoform ratio/balance (Figure 2A). This might be a critical point in oncological correlation analysis since ING splicing isoforms were reported to have different or even opposite functions in tumour cells.

## 4. The Role of ING Isoforms and Splice Variants

Along with different cellular localization, posttranslational modifications, the process of splicing further extends the variety of ING proteins functions [24,60,61]. Alternative promoter usage and alternative splicing is a regulatory mechanism in cells by which one gene gives rise to multiple mature mRNA and proteins varying in their structure and function [62]. Based on current data, all ING family members contain at least two different protein-coding transcripts as a result of alternative splicing or alternative promoter usage, which differ in mass and functional domains constitution (Table 1). In human, the *ING1* gene with three exons gives rise to the alternative variants p33ING1a, p24ING1b, p27ING1c, p47ING1d, and p30ING1e. For the *ING2* gene, two transcripts’ variants were identified *p33ING2a*, and *p28ING2b*, while *ING3* encodes three alternatively spliced isoforms: p11ING3, p47ING3, and the recently identified isoform ING3Δex11 (p43ING3) [38]. *ING4* precursor RNA can be multiply spliced, leading to p29ING4, p28ING4 (isoform 4, ING4_v4), p28ING4 (isoform 1, ING4_v2), p28ING4 (isoform 3, ING4_v3), p25ING4, and p20ING4 proteins. Three different ING5 isoforms were identified and named p28ING5 (isoform 1), p28ING5 (isoform 2), and p26ING5 (isoform 3). p28ING5 (isoform 2) appears as a consequence of the usage of alternate promoter and differs in the 3′ exon structure.

ING proteins possess conserved functional motifs [63]. Further, in addition to alternative splice variants and promoter usage, point mutations can drastically affect the function of the entire protein. For instance, *ING1* missense mutations in the Sin3-associated polypeptide 30 (SAP30) interacting region 102 (R- > L) or at the end of PHD zinc finger codon 260 (N- > S) reduced ING1 activity for nucleotide excision DNA repair in melanoma cells [64]. Missense mutations in the PHD motif of *ING1* and *ING4* abolished their tumour suppressive nuclear function [65,66]. Moreover, other mutations in *ING* genes resulting in protein structure alterations such as protein truncation can lead to more drastic cellular effects. For instance, in mouse mammary glands, the overexpression of a C-terminal truncated ING4, that lacks the PHD motif, resulted in mammary hyperplasia, while co-expression of the mutant *ING4* along with *MYC* oncogene-induced metastasis of breast cancer [67].

Besides mutations, splicing events may change the ING-factor activity. As an example, the lack of functional LZL domain disturbs ING2-induced apoptosis [68], or the splice variant ING3∆ex11 that lacks the PHD cannot prevent EMT induction in PC cells [39]. This is evidence that the splicing of exons from precursor RNA, which results in transcript variants encoding a different composition of functional domains, leads to ING isoforms with modified activity. Thus, ING splice variants need to be taken into special consideration, analysed functionally more precisely, and reveal their cancer type as well as cancer progression-dependent expression. The existence, the level of expression, and the functional analysis of ING2 and ING3 splicing variants likely also applies to PC.

Noteworthy, among all ING1 transcripts, the ING1b isoform represses PC cancerogenesis, since its downregulation is associated with prostate tumorigenesis and affects PC-related signalling pathways [1,69]. Importantly, ING1 isoforms seem to antagonize each other because the ectopic expression of ING1a inhibited ING1b-promoted apoptosis in a dose-dependent manner (Figure 2B) [70,71]. Further, ING1b upregulation was found in senescent human prostate epithelial cells [8,69]. In comparison to the ING1a splice variant, only ING1b seems to be able to bind to the HDAC complex and increases H3 and H4 deacetylation [72,73]; however, both splice variants were shown to induce cellular senescence in other cell types and malignancies, indicating an overlapping pattern of activity. Overexpression of ING1b, the predominantly expressed isoform of ING1 in various human tissues, prompted cellular senescence. The underlying mechanism of ING1b-induced senescence seems to be by association with transcriptional silencing function and interaction with a histone methyl transferase activity [74].

Further, it was reported that the induction of ING1b-mediated cellular senescence is mediated by HAT p300/CBP and their collective binding to the regulatory region of promoter of cell cycle inhibitor p16 and transcriptional induction [35]. The expression level of another ING1 isoform, ING1a, was found to be increased in senescent passages of primary fibroblasts [70]. ING1a induced replicative senescence with characteristic senescent phenotype and upregulated p16 [70], a key cell cycle inhibitor that induces cellular senescence. The known ING1 function in p53 acetylation and p53 stabilisation through a not well-defined mechanism was found to be isoform dependent [75,76,77]. In the study of Liu et al., overexpression of ING1b in p53-positive breast cancer cell line MCF-7 reduced tumour cells proliferation and led to cell cycle arrest in the G0/G1 phase along with p21 upregulation, while the ING1a isoform exhibited no effect on growth nor on cell cycle [78]. Interestingly, only the ING1b and ING1c isoforms, but not ING1a, inhibited hepatoma cell growth by an increased p53 acetylation and indirect stabilization of p53 via interaction with its negative regulator murine double minute 2 (Mdm2) and p14 [79].

Moreover, despite the lack of data about apoptosis regulation by different ING isoforms in PC, ING1 isoforms exhibit also differences in inducing cell death in other cell types. According to Annexin V assay, only ING1b and not ING1a increased the number of apoptotic cells [70]. This is consistent with previous data showing that only ING1b (corresponds to ING1a according to NCBI database) contains PCNA-interacting protein (PIP) domain induced apoptosis through PCNA binding upon UV irradiation, while ING1 proteins lacking PIP domain inhibited UV-mediated cell death [71].

ING3 was also reported to act isoform-specifically in PC. Recently, a novel ING3 splice variant ING3Δex11 was identified in both a human PC cell line and in human PC patient-derived tissues [39]. This new splice variant counteracts the longest ING3 isoform. Interestingly, ING3Δex11 lacks the PHD motif and provokes EMT induction in PC cells [39]. This indicates that the PHD and the binding of ING3 to the histone mark H3K4me3 prevent EMT. In line with this, full-length ING3 with the functional PHD seems to inhibit EMT; therefore, this study showed that PHD domain of ING3 plays a crucial role in EMT suppression in PC cells and two different ING3 isoforms have counteracting behaviour in PC (Figure 2C) [39]. Thus, targeting the ING3 PHD domain to prevent AR coactivator- and chromatin-binding function may not be beneficial.

Analogous to ING3 in PC, ING4 splicing isoforms, missing the full nuclear localization sequence (NLS) (ING4_v2 and ING4_v4), lose tumour suppressive effects, which is rather a characteristic for full-length ING4 (ING4_v1) [80]. Both protein variants, ING4_v2 and ING4_v4, lose the ability to inhibit actin filament polymerization and following cancer cell spreading. ING4_v4 totally loses ING4 full-length function in the suppression of malignant cell migration [80]. Moreover, competition assay between ING4_v1 (full-length ING4) and ING4_v4, using cancer cells transfected with different (ING4 variant-coding) plasmid ratios, showed functional counteraction between splice variants. In detail, ING4_v4 completely reversed the tumour suppressive inhibition of filopodia/lamellipodia formation mediated by ING4 full-length (Figure 2D). In addition, ING4 splice variants with a lack of NLS weakened inhibitory effect on cell growth probably due to their reduced activation of the p21 promoter when compared to ING4 with the intact NLS in three cancer cell lines [80].

Based on these findings, ING splice isoforms arising by mutations or splice variants may differ in the composition of their functional domains and thus can influence basic cellular functions in a distinct manner. Further, they may counteract the activity of wild-type or full-length ING factors, respectively (Figure 2). Hence, the distinct ING isoforms need to be identified, functionally investigated separately, and analysed in the context of a specific cancer type as they were frequently shown to have a tissue-specific function, such as AR coregulator activity in PC. Additionally, some ING splice variants may serve as natural occurring tools to analyse the role of specific ING protein domains. Thus, it is suggested that potential inhibitors of ING factors shall also be analysed for isoform specificity.

## 5. Conclusions

Despite functional and expression analysis by many studies showing tumour suppressive behaviour of ING family members in cancer, some reports demonstrate controversial results. A likely reason for this contradiction might be a cancer-type specificity and/or the expression of newly identified ING splice variants, which differ in their structural protein motifs composition and activity. ING isoforms can counteract and have opposite functions such as tumour suppressive versus oncogenic function in malignancies including PC and therefore require distinct and cancer type-specific analysis.

## Figures and Tables

**Table 1:** ING alternatively spliced variants and ING alternatively used promoter usage isoforms.

ING Factor	ING Isoforms ^1^	Lack of Functional Domain(s) ^2^	Amino Acids	Mass in kDa
ING1	ING1a	-	279	33
	ING1b	PIP, PBD	210	24
	ING1c	PIP, PBD	235	27
	ING1d	PIP, PBD	422	47
	ING1e	PIP, PBD	262	30
ING2	p33ING2a (isoform 1)	-	280	33
	p28ING2b (isoform 2)	LZL	240	28
ING3	p47ING3 (isoform 1)	-	418	47
	p43ING3 (ING3Δex11)	PHD	405	43
	p11ING3 (isoform 3)	NLS, PHD	92	11
ING4	p29ING4 (isoform 9, ING4_v1)	-	249	29
	p28ING4 (isoform 4, ING4_v4)	NLS	245	28
	p28ING4 (isoform 1, ING4_v2)	NLS	248	28
	p28ING4 (isoform 3, ING4_v3)	NLS	246	28
	p25ING4 (isoform 5, ING4Δex2)	LZL, NLS	225	25
	p20ING4 (isoform 6, ING4ΔEx6A)	PHD	179	20
ING5	p28ING5 (isoform 1)	-	240	28
	p28ING5 (isoform 2)	PHD	254	28
	p26ING5 (isoform 3)	PHD	226	26

^1^ Note that the nomenclature of ING isoforms is according to the NCBI protein database, which may differ in some previous publications. ^2^ Abbreviations: LZL (leucine zipper-like), PBD (partial bromodomain), PIP (PCNA-Interacting Protein Motif), NLS (nuclear localization sequence), and PHD (plant homeodomain).

## Data Availability

Supplementary Figure S1: The data were obtained from http://ualcan.path.uab.edu/analysis.html (accessed on 23 August 2021); for Supplemental Figure S2: http://ualcan.path.uab.edu/analysis.html (accessed on 23 August 2021).

## Supplementary Materials

The following are available online at https://www.mdpi.com/article/10.3390/cells10102599/s1, Figure S1: lack of significant association between ING expression levels of non-tumour prostate and PC specimens, Figure S2: lack of significant correlation between ING expression levels in PC tissues and patient’s survival

## Abbreviations

AR, androgen receptor; DBD, DNA-binding domain; EMT, epithelial-mesenchymal transition; HAT, histone acetyl transferase; HDAC, histone deacetylase; ING, inhibitor of growth; NLS, nuclear localization signal; PC, prostate cancer; PHD, plant homeodomain.
