# Supplementary material for: ING Tumour Suppressors and ING Splice Variants as Coregulators of the Androgen Receptor Signalling in Prostate Cancer
Source: Cells. 2021 Sep 29;10(10):2599. doi: 10.3390/cells10102599 (PMC8533759; doi:10.3390/cells10102599)
Supplement: Supplementary file 1 [file cells-10-02599-s001.zip › cells-1399599-supplementary.pdf]

Supplemental figures

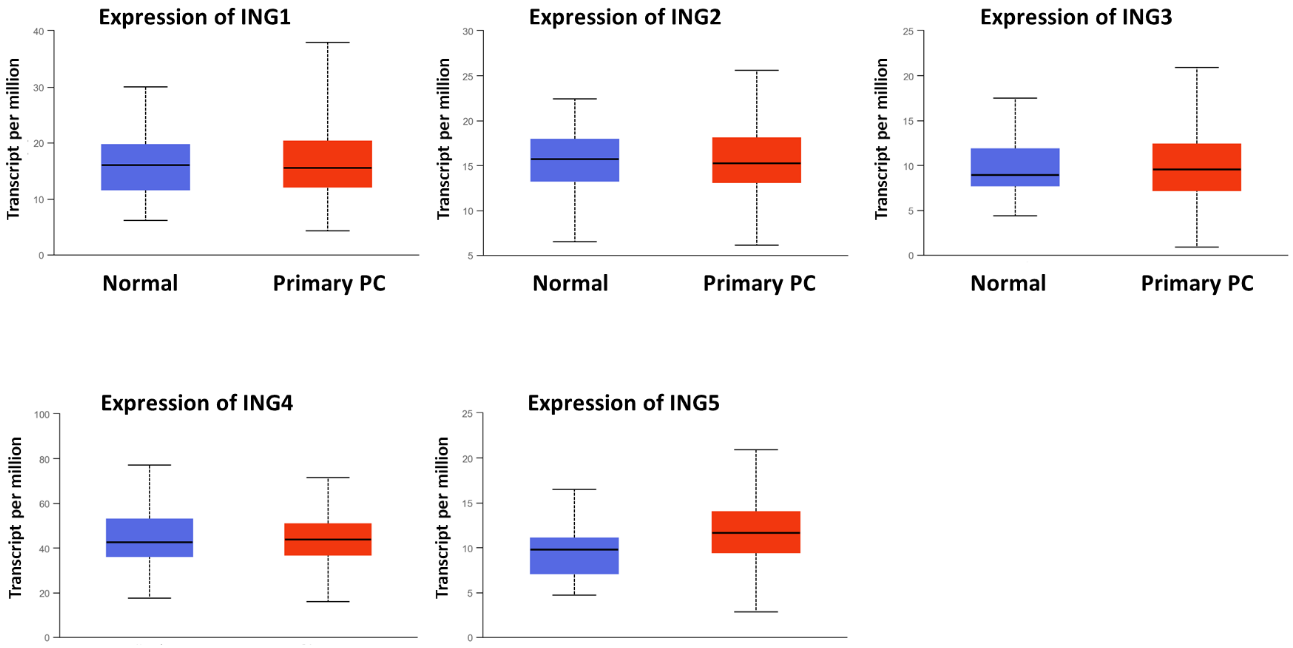

Figure S1. Lack of significant association between ING expression levels of non-tumour prostate and PC specimens. UALCAN database expression analysis of 52 normal prostate tissue samples (n=52) and 497 PC samples (n=497) from the cancer genome atlas (TCGA) dataset in PRAD-based sample types. Blue bar represents normal samples, red one represents PC (primary tumour) samples. Please note, that ING isoforms are not considered. The solid black line is median. The data was obtained from http://ualcan.path.uab.edu/analysis.html on 23.08.2021.

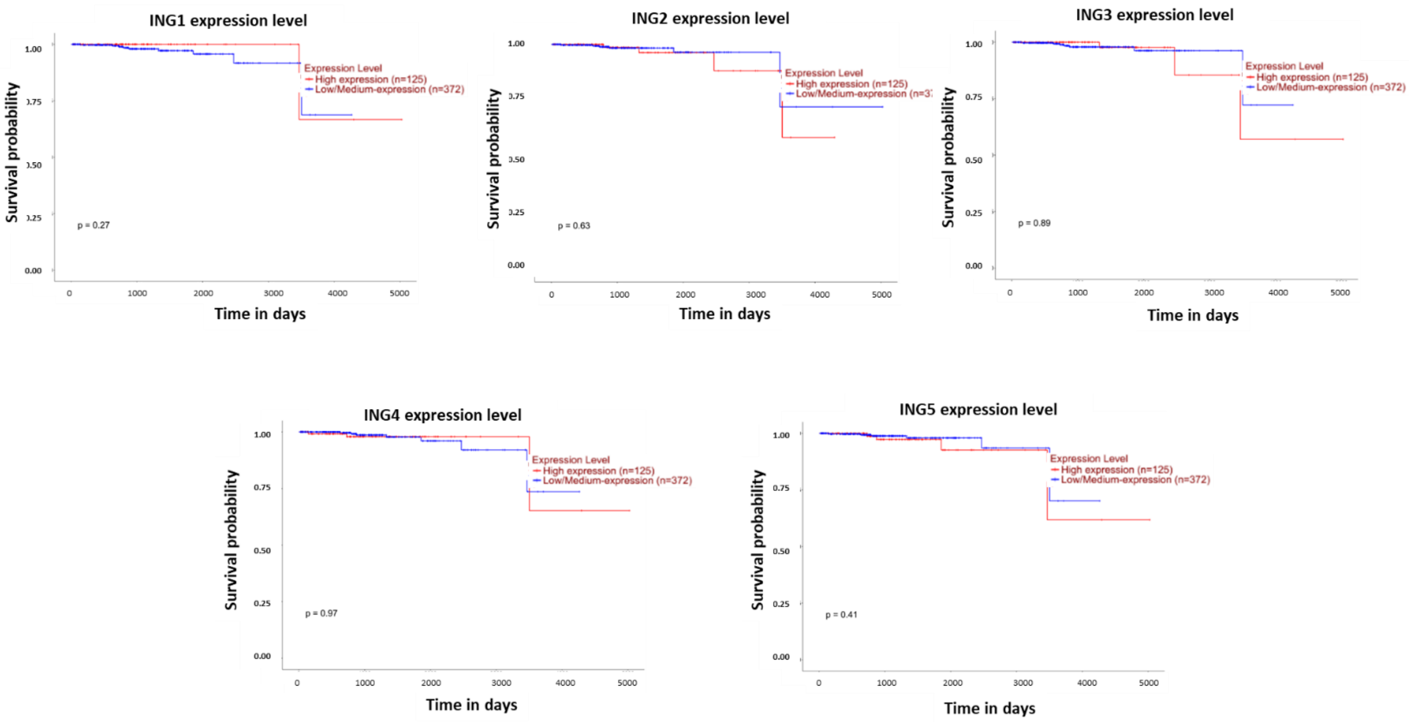

Figure S2. Lack of significant correlation between ING expression levels in PC tissues and patient's survival. UALCAN database expression analysis of 125 PC patients with high ING expression in PC tissue samples (n=125) and 372 low ING expression in PC samples (n=372) from the cancer genome atlas (TCGA) dataset in PRAD-based sample types. Blue line represents samples with low ING expression, red line represents high ING expression in PC. The data was obtained from http://ualcan.path.uab.edu/analysis.html on 23.08.2021.
